# Recurrent intermittent hyponatremia: A new experimental model

**DOI:** 10.1371/journal.pone.0341743

**Published:** 2026-02-20

**Authors:** Marta Tejedor, Lorena Cussó, María Ángeles González-Nicolás, Diego San Felipe, Daniel Calle, Beatriz Martín-Sánchez, Baris Uzun, Giovanna Martín-Palumbo, Luis Antonio Alvarez-Sala Walther, Eva M. Marco, Meritxell López-Gallardo, Alberto Lázaro, Manuel Desco

**Affiliations:** 1 Hepatology, Department of Gastroenterology and Hepatology, Hospital Universitario Infanta Elena, Valdemoro, Madrid, Spain; 2 Department of Medicine, School of Medicine, Universidad Complutense, Madrid, Spain; 3 Renal Physiopathology Laboratory, Department of Nephrology, Instituto de Investigación Sanitaria Gregorio Marañón (IiSGM), Hospital General Universitario Gregorio Marañón, Madrid, Spain; 4 Department of Internal Medicine, Gastroenterology and Hepatology, University of Iowa, Iowa City, Iowa, United States of America; 5 Unidad de Medicina y Cirugía Experimental, Instituto de Investigación Sanitaria Gregorio Marañón, Madrid, Spain; 6 Unidad de Imagen Avanzada, Centro Nacional de Investigaciones Cardiovasculares (CNIC), Madrid, Spain; 7 CIBER de Salud Mental, Instituto de Salud Carlos III, Madrid, Spain; 8 Department of Physiology, School of Medicine, Universidad Complutense, Madrid, Spain; 9 Department of Genetics, Microbiology and Physiology, Faculty of Biological Sciences, Universidad Complutense de Madrid, Madrid, Spain; 10 Departamento de Bioingeniería, Universidad Carlos III de Madrid, Leganés, Madrid, Spain; Albany Medical College, UNITED STATES OF AMERICA

## Abstract

**Aim:**

It is assumed that natremia remains low throughout the duration of chronic hyponatremia, inducing symptoms. We explore Recurrent Intermittent Hyponatremia (RIH) to establish whether a few hours of hyponatremia per day are enough to induce significant water retention.

**Methods:**

A rat model of RIH was developed and compared to control animals. Electrolyte balance and central nervous system (CNS) (water content and cellular changes) were studied at baseline and after a water overload equivalent to 10% of the animal’s body weight. Apparent diffusion coefficient (ADC) obtained through magnetic resonance was used to study changes in content and distribution of brain water. Immunohistochemistry techniques were used to study in the CNS astroglial cells through the glial fibrillary acidic-protein (GFAP) expression; as well as oligodendrocytes and the myelin sheath using the myelin basic protein (MBP) antibody. Blood and urine analysis were performed to assess water and electrolyte balance.

**Results:**

In RIH, mild hyponatremia was induced, but recovered after 24h; this situation was repeated for 7 consecutive days. A lower ADC value in the whole brain compared to control animals suggested an increase in total brain water in this situation. An increase in GFAP expression in the gray matter (GM) was observed, while MBP’s expression remained unchanged. The water overload on RIH induced hypotonic hyponatremia, progressively decreased ADC values in the whole brain (less pronounced than in controls), and increased GFAP and MBP’s expression in the white matter (similar increase found in controls).

**Conclusion:**

RIH is a novel animal model that suggests there can be significant water retention after only a few hours of hyponatremia a day provided this situation is repeated over time. Such water retention translates into a greater brain water accumulation and astroglial activation in the GM.

## Introduction

Hyponatremia is defined as a serum sodium concentration (Na^+^_p_) lower than 135 mEq/L. The vast majority of hyponatremias are hypoosmolar, associated with plasma osmolalities lower than 275 mOsm/kg. Water balance is impaired, leading to water retention generally as a consequence of elevated antidiuretic hormone (ADH) levels. There is no sodium deficit *per se* [1].

Hyponatremia is the most common electrolyte disorder in clinical practice, affecting up to 5% of adults in the general population. Prevalence increases with age, reaching 20% among patients over 65 years of age, and up to 40% of in-patients may develop this condition [2]. Most cases are mild (Na^+^_p_ 130–135 mEq/L) [2–4], but even in mild forms, hyponatremia is associated with worse prognosis and higher overall mortality than normonatremia [5–8].

Clinical manifestations depend not only on the magnitude of the decrease in serum sodium concentration but also on the rapidity of onset, with severe symptoms (seizures, cardio-respiratory compromise, brainstem herniation or respiratory arrest) more likely developing in acute hyponatremia [1,7,9]. Chronic mild hyponatremia has been shown to be associated with attention deficits, gait instability and impaired motor function, increased latency in solving tasks and increased error rates, and an increased risk of fractures [10–12]. These symptoms are a consequence of water accumulation inside brain cells [13–17], leading to a volumetric increase of the encephalic tissue and cerebral edema in situations of hypoosmolality. As the brain is contained in a rigid structure, the skull, adaptation mechanisms have developed to prevent fatal complications. In the first few minutes, excess water in the extracellular encephalic space is transferred to the cerebrospinal fluid (CSF) in favor of the hydrostatic gradient to reduce intracranial pressure [18,19]. Afterwards, astrocytes extrude osmotically active substances that draw water into the extracellular space, decreasing their cell volume through a process termed regulatory volume decrease. Consequently, the global encephalic volume is maintained. In the first 3–24 hours, electrolytes (Na^+^, K^+^, Cl^-^) and water are excreted, in the so-called rapid adaptation [20,21]. If hyponatremia persists over time, a slow adaptation with extrusion of organic osmolytes will occur [22]. In this situation where the brain has adapted to a low natremia, patients may present with worsening neurological symptoms if their serum sodium concentration drops further acutely.

Water intoxication allows easy modelling of acute hyponatremia. Hypotonic solutions, mainly distilled water or 5% dextrose water, have been administered intraperitoneally (i.p.) in volumes equivalent to 10–40% of the body weight of experimentation animals over variable periods of time (as a bolus or over several hours), and associated or not with the administration of a single dose of desmopressin acetate (ddAVP), a structural analogue of ADH that favors renal water retention. This approach is the most widely used in rodents [5,10,18–31].

The development of animal models for the study of chronic hyponatremia has been more complex. Early murine models of chronic hyponatremia kept the animals fasted, and associated daily injections of an artificial ADH derivative, pitresin tannate (no longer marketed) that induced renal water retention [32]. Shortly thereafter, Verbalis et al.[33,34] generalized the use of water restriction in combination with continuous ddAVP infusion using subcutaneous pumps in rodents for the study of moderate-to-severe hyponatremia sustained over time. However, it is rare to find in human clinical practice sustained severe hyponatremia such as those reproduced with these models.

What has not been studied so far is intermittent but recurrent hyponatremia, and therefore its relevance is not known. There are scenarios in human clinical practice where this situation could occur and go unnoticed, such as decompensated cirrhosis or heart failure, where patients have high baseline ADH levels and often receive diuretic treatment, which can cause baseline natremia to be in the lower limit of normal [35,36]. In this situation, any excess in water intake could induce transient mild hyponatremia of only a few hours’ duration that could recover prior to the next blood test. The clinical significance of this theoretical framework has not yet been studied.

Therefore, our objective is the development of a rat model of “Recurrent Intermittent Hyponatremia” (RIH), in which animals are intermittently injected with ddAVP and water, using a hyposodic diet. We explore the changes that this condition could induce on water and electrolyte balance, as well as on cells and water content in the central nervous system. We hypothesized that mild RIH would be enough to induce significant water retention in experimental animals. In that case, animals with RIH would also be more susceptible to significant worsening in the event of an acute water overload (similar to what is observed in clinical practice with acute worsening of chronic hyponatremia).

## Materials and methods

### Animals and experimental protocols

Studies were carried out on female Wistar rats (Charles River, Wilmington, MA, USA), with an average weight of 182.01 ± 34.51 g. Rats were housed in the animal facility of the Hospital General Universitario Gregorio Marañón (HGUGM), Madrid, Spain (ES280790000087). The experimental protocol was conducted in compliance with Spanish legislation (Royal Decree 53/2013, 23^rd^ February1) and European Directive (2010/63/EU) on the protection of animals used for experimentation and other scientific purposes, and was approved by the HGUGM Animal Experimentation Ethics Committee, the local Ethics Committees, and the Animal Protection Board of the Comunidad Autónoma de Madrid (protocol codes: PROEX 059/15 and 129.2/21).

All animals were housed in individual plastic cages in a temperature (24ºC) and humidity- controlled room with lights on from 8 a.m. to 8 p.m. and 5–6 air renovations per hour. Weight, water and food consumption were measured daily for all animals.

#### Experiment 1: Establishment of Recurrent Intermittent Hyponatremia.

Four animals constituted the RIH group receiving a hyposodic jellified liquid diet (Rodent Liquid Diet F 1268SP BioSerV, New Jersey, USA). It was necessary to jellify the hyposodic diet to ensure adequate ingestion by the animals, and after several edibility trials at different concentrations, the best results were obtained by diluting this diet 4 times with respect to the manufacturer’s recommendations. After one week on the hyposodic diet, animals spent 24h in a metabolic cage (ref. 526756, Harvard apparatus®, USA) to collect and analyze the urine. The following morning, a blood sample from the tail vein was obtained to assess the effect of the change in diet on the internal *milieu.* The following week, these animals received daily i.p. injections of ddAVP (Minurin®, Ferring, Madrid, Spain) at a dose of 0.4 µg/kg, along with an i.p. volume of distilled water equivalent to 2.5% of the animal’s body weight, for 7 days (ddAVP/water). The dose of ddAVP used in the experiments was based on human clinical practice, adjusted by weight. A blood sample from the tail vein was obtained daily 4:30h after the injections.

The RIH group was compared to a control group (n = 4) receiving standard pellet based diet (EU Rodent Pellet Diet 14% 5LF2 LabDiet, St Louis, USA). The amount of calories offered to all animals was similar, as the amount of pellet chow was restricted to pair the calories in the hyposodic diet. Both groups had *ad libitum* access to water throughout the experiment.

At the end of the second week, all animals spent 24h in a metabolic cage to collect and analyze the urine. The following morning, under deep sedation with 80 mg/kg ketamine (Ketolar® 50 mg/ml, Parke-Davis SL, Pfizer group, Madrid, Spain) and 10 mg/kg xilacin (Xylasol 20 mg/ml, Laboratorios Karizoo S.A., Alvira group, Barcelona, Spain), sacrifice was undertaken by direct heart puncture to obtain a blood sample.

To study the brain effects of the RIH model we compared baseline MRIs (before water overload) of controls and RIH obtained in experiment 2.

#### Experiment 2: Acute hyponatremia and acute hyponatremia on Recurrent Intermittent Hyponatremia.

A second experiment explored the effect of an acute water overload on controls (induction of acute hyponatremia) and RIH (induction of acute on RIH) animals. A single i.p. injection of distilled water equivalent to 10% of the animal’s body weight (water overload) was administered on day 15–4 animals fed for two weeks with a pellet-based diet, and to 4 animals submitted to RIH (one week on a hyposodic diet followed by another week of daily i.p. administration of ddAVP/water). Prior to the administration of the water overload, a retro-orbital blood sample was taken under anesthesia with sevofluorane (5% for induction and 2% for maintenance, in 100% oxygen), and a baseline magnetic resonance image (MRI) was obtained (see below). Two hours later, after completion of the MRI study and still under sedation, a second blood sample was taken immediately before sacrifice. During sacrifice, a urine sample was collected by bladder puncture.

To rule out any possible effect of anaesthesia on physiological parameters [37] we performed the same MRI sedation protocol on two pellet-fed animals. Animal’s heart rate (HR), oxygen saturation (SatO2), respiratory rate (RR) and temperature (Tª) were recorded every 10 minutes for 120 minutes by MRI compatible module (S.A. instruments, INC). No significant changes were observed as a result of the sedation protocol in the longitudinal recording of animal’s vitals (data not shown), except for a significant increase in pO2 (pre: 74.50 ± 2.12 vs post 136.00 ± 11.31 mmHg, p = 0.017), possibly due to the use of supplemental oxygen administered along the anaesthesia during this period.

### Central nervous system sample collection

After the MRI study, rats were sacrificed with an overdose of ketamine and xilacin. Rats were transcardially perfused with saline solution (NaCl 0.9%, B. Braun Medical SA) with 0.001% heparin at 4ºC with a volume of 120−150 ml, and then with a 4% paraformaldehyde (PFA, Merck) solution in 0.1 M phosphate buffer (PB) at pH 7.4and 4 °C as a fixative solution (same volume as with the first solution). After perfusion, the brains were quickly removed and immersed in a 4% PFA solution for 48h at 4ºC. Then, brains were washed 3 times with 0.1M PB (pH 7.4) of 30 min each. Tissue was cryoprotected in 11% sucrose in 0.1M PB at 4°C for 24h, and then in 33% sucrose in 0.1M PB at 4°C for a further 48h period. Subsequently, brains were placed in a tissue-freezing medium (Tissue-Tek® O.C.T. Optimal cutting temperature, Sakura®, Finetek USA Inc, Torrance, CA, USA), and stored at −30ºC until use. The brains were frozen sectioned using a cryostat (LEICA biosystems CM3050, Deer Park, IL, USA) into 20 μm coronal sections. Tissue sections were collected into four alternate series of gelatin-coated slides (4 slices per slide), air dried and stored until use at −30°C.

### Biochemical analyses

Direct measurement of serum Na^+^, K^+^, glucose and haematocrit were performed using a GEM Premier 3000 (Instrumentation Laboratory, Werfen, Barcelona, Spain) gas analyser. Blood and urine osmolality were measured using an osmometer (Fiske one-ten osmometer, Fiske associates, MA, USA). Urinary ions were measured using an auto-analyzer CObAS711 (Roche Farma, Madrid, Spain). 24h diuresis was measured during the experiment 1 using metabolic cages (see Material and Methods; Animals and Experimental Protocols; Experiment 1: Establishment of Recurrent Intermittent Hyponatremia for details).

From these direct measurements obtained, a series of renal function parameters were calculated [38]:


$$\text{Urinary sodium excretion}\;:\left(\text{Urine volume}*\;\text{Urinary }{\text{Na}}^{+}\right)\;/\text{1000 mEqd}$$

$$\text{Urinary potassium excretion}\;:\left(\text{Urine volume}\;*\;\text{Urinary }{\text{K}}^{+}\right)\;/\text{1}\text{0}\text{0}\text{0}\text{ mEqd}$$

Free water clearance:Urine volume*(1−Urinary Osmolality/Plasmatic Osmolality) mL/d

### Evolution of weight, and caloric and water intake

Daily weight progression was assessed during the week of adaptation to the different diets, and subsequently during the week of ddAVP/water administration. The daily intake of water and calories was measured during the same periods of time, from which the intake of sodium and potassium was calculated using the diet composition as a reference.

### Magnetic resonance imaging studies

To study the brain effects of RIH and acute water overload, MRI brain studies were acquired with a 7 Tesla BioSpec 70/20 scanner (Bruker, Ettlingen, Germany) in anesthetized (5% sevoflurane in 100% oxygen) rats. Diffusion Weighted Images (DWI) were acquired using a single-shot planar echo sequence with the following parameters: repetition time (TR) = 1500 ms, echo time (TE) = 20.2 ms, 10 slices, slice thickness 1 mm, axial orientation, field of view of 27 x 32 mm^2^ and matrix size of 192 x 92 pixels. Five different b values (0, 49, 308.8, 1039.8, 2026 s/mm^2^) in the X direction (transverse to the magnetic field) were used to obtain five different images which were used to calculate apparent diffusion coefficient (ADC) maps. The study comprised a total of thirteen DWI acquired every 10 minutes for 120 minutes. Immediately after baseline imaging (time 0), all animals received an i.p. bolus of distilled water equivalent to 10% of the animal’s weight, with the aim of inducing acute hyponatremia (pellet-diet) or acute hyponatremia on RIH (hyposodic-diet + ddAVP/water).

To calculate ADC values, regions of interest (ROIs) were manually defined using ImageJ software (version 1.52a, National Institutes of Health, NIH, USA) in 2–3 consecutive axial slices, except for whole brain ROI, where all axial slices were used. The ROIs chosen included grey matter (GM) (caudate-putamen, cortex, hippocampus), hypothalamus, white matter (WM) (corpus callosum, anterior commissure, internal capsule) and whole brain. This latter value was measured both with and without the contribution of CSF. The subregions included in the GM were also analyzed separately. For the localization and delimitation of ROIs, axial slices from the Paxinos murine neuroanatomy atlas [39] were used as reference (Bregma cortex −3.10 to −3. 68 mm; corpus callosum, anterior commissure and internal capsule Bregma −4.10 to −4.74 mm; caudate-putamen Bregma −4.60 to −6.10 mm; hippocampus Bregma −6.38 to −7.60 mm; hypothalamus Bregma −8.82 to −9.22 mm).

Also 1H spectra of the caudate-putamen were acquired prior to the i.p. bolus injection of water (10% of body weight) using a PRESS sequence (FOV: 1.3x1.5x1.5 mm^3^; TE = 16.5 ms, TR = 2500 ms, 256 averages, 2048 acquisition points and a bandwidth of 3001 Hz). The osmolytes quantified were glutamine/GABA, myoinositol, choline, N-acetyl aspartate (NAA) and creatine. Metabolite peak areas were normalized to the water peak, in order to obtain values proportional to the real metabolite concentration instead of just ratios to creatine concentration. Quantification was performed using Mnova software (Mestrelab Research, Santiago de Compostela, Spain).

Pre- and post-assessment retro-orbital blood sample were drawn, as above. At the end of the image acquisition period, and still under deep sedation, the animals were sacrificed and the brain extracted.

### Immunohistochemical analyses of astrocytes and oligodendrocytes

Immunohistochemical analyses were performed according to a protocol published elsewhere [40]. To evaluate astroglial cells a primary antibody for glial fibrillary acidic-protein (GFAP) was used (rabbit anti-GFAP, 1:600 dilution, Ref. Z0334 Dako Agilent, Santa Clara, CA, USA); and a goat anti-rabbit IgG (H + L) biotinylated (Ref. 31820 Thermo Scientific, Waltham, MA, USA) 1:200 dilution, was employed as secondary antibody. To assess oligodendrocytes and myelin sheath a primary anti-myelin basic protein (MBP) antibody was used (mouse anti-MBP, 1:200 dilution, Ref. SC-271524, Santa Cruz Biotechnology Inc, Dallas, TX, USA), and a secondary goat anti-mouse IgG (H + L) biotinylated (BA-9200, Vector Laboratories, Newark, CA, USA) antibody, 1:200 dilution, was used. The slides with the samples were incubated for 1.5h with ABC-peroxidase complex (Streptavidin-Biotin Complex-Peroxidase, Thermo Scientific Waltham, MA, USA), 1:250 dilution. The reaction product was revealed by incubating the sections with 0.45 mg/mL of 3, 3’-diaminobenzidine tetrahydrochloride hydrate (Sigma-Aldrich, USA, Ref. D5637) and 0.03% H_2_O_2_ in PBS. Finally, sections were coversliped with the mounting medium Depex (Serva®). Each immunohistochemistry assay contains slides from the four animals of each experimental group (RIH [hyposodic diet + ddAVP/water], acute on RIH [hyposodic diet + ddAVP/water + water overload] and acute hyponatremia [pellet diet + water overload] models); and a negative control (without adding primary antibody) to check the specificity of the immunoreaction.

Immunostained slides were observed under a Zeiss AxioImagerA.12 microscope (Zeiss Axioplan Microscope, Oberkochen, Germany), linked to a high-resolution camera (Zeiss Axioplan 712 color, Germany), which was used for capturing the images. Image processing was performed in ZEN3.3 software, version 3.3.89.0000 (Carl Zeiss AG, Oberkochen, Germany). Magnification, light, shine and contrast conditions were kept constant during the capture process. Figures were prepared using Adobe Photoshop CS4 Extended 10.0 (Adobe Systems, San Jose, CA, USA).

#### Quantitative evaluation.

Immunostaining data were analysed on microphotographs at 10x magnification. The brain regions evaluated were motor cortex, corpus callosum, internal capsule and periventricular hypothalamus. Neuroanatomical structures were identified using the Paxinos brain atlas [39]. The anterior–posterior (AP) localization from bregma of the analyzed regions was as follows: cortex and anterior corpus callosum Bregma 2.52 to 1.92 mm; hypothalamus and medial corpus callosum Bregma −0.96 to −1.44 mm; internal capsule and posterior corpus callosum Bregma −2.64 to −3.24 mm. For each brain structure, one slice was randomly selected from each slide. As the structures evaluated are bilateral, the mean values between hemispheres were used. The included regions were analyzed separately and subsequently grouped into GM (motor cortex, hypothalamus) and WM (corpus callosum, internal capsule).

Quantification of GFAP and MBP expression was performed by optical densitometry of the microphotographs with the ImageJ program, using ROIs for each brain structure analyzed obtaining an optical density value (arbitrary units). The area measured with the ImageJ program in pixels was converted to µm^2^ taking into account that each pixel corresponds to 0.6573 µm^2^ (S1 Table).

### Data analysis

Quantitative variables are presented as mean ± standard deviation (SD) while qualitative variables are expressed as frequencies and percentages.

For analyses of continuous variables versus independent factors, a factorial analysis of variance (ANOVA) was applied. These data satisfied the assumptions of normality and homogeneity of variances (Kolmogorov and Levene tests). The information provided is the significance of the model, the significance of each factor and, whenever necessary, the significance of the interaction between factors. Differences between factor levels were investigated using post hoc tests (Tukey and Fisher’s least significant difference (LSD)).

ADC values comparison between groups by region, and their evolution over time, was studied using linear mixed models. Animals were considered as a random effect, while group, time, and group-time interaction were considered as fixed effects.

Statistical significance threshold was set at a P value of <0.05.

All statistical analyses were performed with IBM SPSS Statistics for Windows, Version 25.0. Armonk, NY: IBM Corp.

## Results

### Establishment of recurrent intermittent hyponatremia

a) **Internal *milieu***

Four animals were fed with a jellified hyposodic diet for one week. At the end of this period the animals had lost weight (initial 239.50 ± 17.06 vs final 227.50 ± 13.08 g, p = 0.077). Calories ingested daily with the hyposodic diet were on average lower than with pellets (40.10 ± 12.85 vs 56.08 ± 10.06 cal/d, p < 0.001). The change in diet did not induce changes in natremia or plasma osmolality. Water intake, as well as sodium and potassium intake, was lower on the hyposodic diet (Table 1). In addition, animals on the hyposodic diet showed a marked increase in diuresis and free water clearance (Table 1), along with a reduction in urinary concentration (pellet 736.00 ± 145.79 vs hyposodic 189.25 ± 32.65 mOsm/kg, p < 0.001).

**Table 1:** Comparison of analytical values between pellet diet hyposodic diet and Recurrent Intermittent Hyponatremia (RIH).

Variable	Pellet diet	Hyposodic diet	RIH
Natremia(mEq/L)	140.25 ± 3.30	136.50 ± 1.73	141.25 ± 0.96
Serum osmolality(mOsm/kg)	302.67 ± 11.55	310.00 ± 10.71	289.33 ± 2.31 ^$^
Water intake(mL/d)	29.25 ± 5.72	13.96 ± 3.33 *	25.92 ± 9.11 ^$^
Na^+^ intake(mEq/d)	1.66 ± 0.11	0.69 ± 0.16 *	0.49 ± 0.19 *^$^
K^+^ intake(mEq/d)	2.21 ± 0.14	0.23 ± 0.05 *	0.16 ± 0.06 *^$^
Diuresis(ml/d)	13.75 ± 2.10	72.25 ± 11.70 *	50.00 ± 23.22 *^$^
Urinary osmolality(mOsm/kg)	736.00 ± 145.79	189.25 ± 32.65 *	201.25 ± 48.37 *
Urinary Na^+^ excretion(mEq/d)	0.86 ± 0.15	1.04 ± 0.31	0.74 ± 0.40
Urinary K^+^ excretion(mEq/d)	0.92 ± 0.20	0.49 ± 0.20 *	0.27 ± 0.21 *
Free water clearance (mL/d)	−20.30 ± 4.79	28.55 ± 10.95 *	10.00 ± 1.42 *^$^

Results expressed as mean ± SD; n = 4 per group. * p < 0.05 compared to pellet diet. $ p < 0.05 compared to hyposodic diet. Na^+^: sodium. K^+^: potassium.

**Fig 1:**
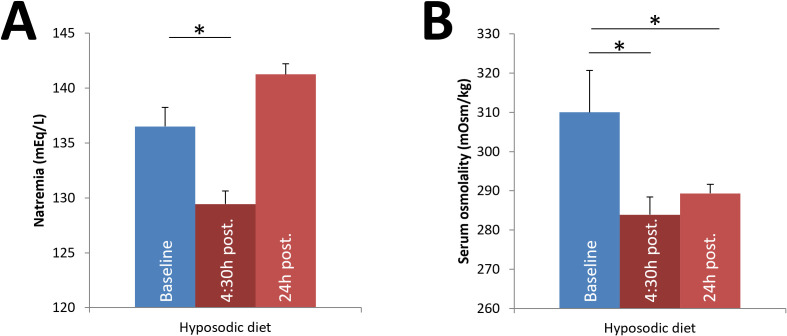
Evolution of natremia and serum osmolality in the first 24h after intraperitoneal (i.p.) administration of ddAVP/water in the hyposodic diet group. Columns show mean baseline values; mean values of all measurements at 4.5h after the daily injection of ddAVP/water; and final mean value 24h after the last injection. Results are expressed as mean ± SD; n = 4 per group. *p < 0.05 for comparison vs. baseline.

The effect of daily i.p. administration of ddAVP/water to the hyposodic diet group (hyposodic diet + ddAVP/water) was assessed. Natremia was measured every day 4:30h after the morning injection of ddAVP/water (baseline Na^+^_p_ 136.50 ± 1.73 vs post-injection 129.44 ± 1.20 mEq/L, p < 0.001; baseline Osm_p_ 310.00 ± 10.71 vs post-injection 283.91 ± 4.52 mOsm/L, p = 0.030). However, 24h after the last dose of ddAVP/water, serum sodium returned to normal (Na^+^_p_ 141.25 ± 0.96 mEq/L, N.S. vs. baseline), but plasma osmolality remained below the baseline value (289.33 ± 2.31 mOsm/L, p = 0.024) (Fig 1). Glucose levels were significantly lower in the RIH group (140 ± 11.34 vs 192.67 ± 14.74 mg/dL, p = 0.003).

After administering ddAVP/water to animals on a hyposodic diet, there was no statistically significant change in animal’s weight (initial 223.50 ± 12.07 vs final 210.00 ± 17.15 g, N.S.) despite a trend towards reduced daily caloric intake compared to hyposodic diet alone (40.10 ± 12.85 vs 32.39 ± 18.88 cal/d, p = 0.081). Water intake increased significantly compared to baseline, while diuresis and free water clearance significantly reduced compared to baseline (Table 1).

Therefore, the i.p. administration of ddAVP/water did not induce chronic hyponatremia, but did produce RIH, a mild and transient hyponatremia that recovered 24h after the administration of ddAVP/water, a situation that was repeated over several consecutive days. These data suggest the existence of a net water retention even though chronic hyponatremia was not induced. We then explored a possible increase in cerebral edema in this newly described condition.

b) **Brain changes on MRI**

**Fig 2:**
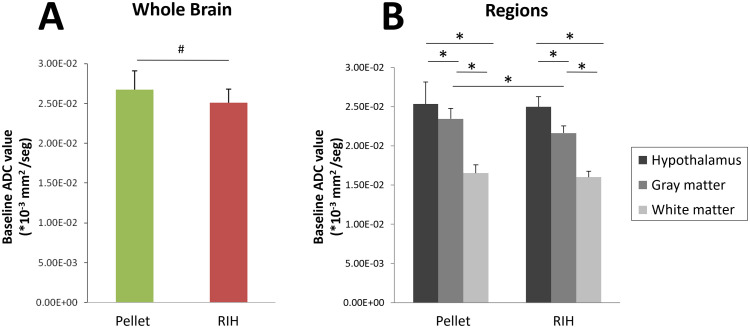
Baseline values of the apparent diffusion coefficient (ADC), on day 15 of the study protocol, prior to the acute water overload. Panel A: comparison of the basal ADC value in the whole brain between the pellet fed group and the recurrent intermittent hyponatremia (RIH) group (hyposodic diet + ddAVP/water). Results expressed as mean ± SD; n = 4 per experimental group. #p < 0.05 between pellet diet and RIH. Panel B: comparison of baseline ADC values between different brain regions in the different experimental groups. Results expressed as mean ± SD; n = 4 per group.*p < 0.05 between the different regions studied in each one of the groups. In the comparison between the regions in both experimental groups, ADC was lower in the gray matter in the RIH group (p = 0.054); this has been represented in the figure as statistically significant.

On day 15 of the study protocol, 24h after the last ddAVP/water administration to the RIH group, and prior to any water overload, baseline ADC values were studied in both groups. In the RIH group the ADC value for the whole brain was 25.07 ± 1.71 mm^2^*10^3^/sec, lower than in the control group (26.71 ± 2.40 mm^2^*10^3^/sec, p = 0.05) (Fig 2A). Similar results were observed when CSF contribution was discounted (whole brain without CSF in RIH was 23.1 ± 1.06 vs 24.29 ± 1.66 mm^2^*10^3^/sec in the control group, p = 0.043) (S2 Table). ROI analysis showed that ADC value in GM was higher than that of WM (21.63 ± 0.95 vs. 16.02 ± 0.75 mm^2^*10^3^/sec, p < 0.001) (Fig 2B) in the RIH group, similarly to what happened in the untreated pellet-fed group (GM 23.45 ± 1.33 vs. WM 16.52 ± 1.07 mm^2^*10^3^/sec, p < 0.001). ADC values in the GM were marginally lower in animals submitted to RIH compared to those pellet-fed (p = 0.054), and no significant differences were found between both groups in the WM. The individual values of regions analyzed are available in S2 Table. *In vivo* spectroscopic analysis showed no significant differences between the RIH and the untreated pellet groups in myosin, choline, glutamine/gamma-aminobutyric acid (GABA) or N-acetylaspartate (NAA).

c) **Brain changes on immunohistochemical analyses**

**Fig 3:**
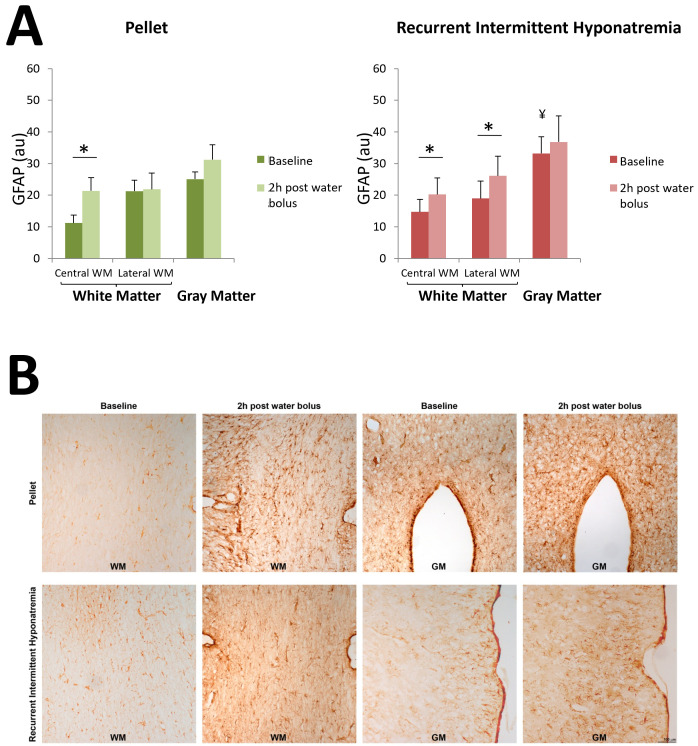
Effects of an intraperitoneal (i.p.) water overload equivalent to 10% of the animal’s body weight on the expression of glial fibrillary fibrillary acidic protein (GFAP) compared to baseline (on day 15 of the study protocol, prior to the acute water overload). Results are expressed in arbitrary optical density units (au) as mean ± SD; n = 4 per experimental group. Panel A left: Pellet-fed animals. Factorial ANOVA of model p < 0.001; factor water bolus p < 0.001; factor region (central WM/lateral WM/GM) p < 0.001; interaction water bolus*region (central WM/lateral WM/GM) p < 0.001. *p < 0.05 for the comparison of central WM before and after water bolus administration. Panel A right: Recurrent intermittent hyponatremia (RIH) group (hyposodic diet + ddAVP/water). Factorial ANOVA of model p < 0.001; water bolus factor p < 0.001; region factor (central WM/lateral WM/GM) p < 0.001; interaction water bolus*region (central WM/lateral WM/GM) N.S. *p < 0.05 for the comparison of central WM before and after a water bolus, and for the comparison of lateral WM before and after administration of a water bolus. ¥ p < 0.05 for the comparison of GM at baseline between pellet-fed animals and RIH. Panel B: The images show: WM (corpus callosum) in pellet fed animals at baseline and 2h post water bolus; GM (hypothalamus) in pellet fed animals at baseline and 2h post water bolus; WM (internal capsule) under RIH at baseline and 2h post water bolus; and GM (motor cortex) under RIH at baseline and 2h post water bolus. WM: white matter. GM: gray matter. RIH: recurrent intermittent hyponatremia.

On day 15 of the study protocol, 24h after the last ddAVP/water administration to the RIH group, and prior to any water overload, baseline immunohistochemical analysis was performed in both groups. Immunohistochemical analysis of astrocytes showed an increased GFAP expression in GM compared to WM, both in the RIH and the pellet-fed groups (p < 0.001 for both groups). Compared to untreated pellet-fed animals, the induction of RIH increased GFAP expression in the GM (p = 0.031) (Fig 3). Optical density values obtained for each of the regions studied separately are available in S3 Table.

**Fig 4:**
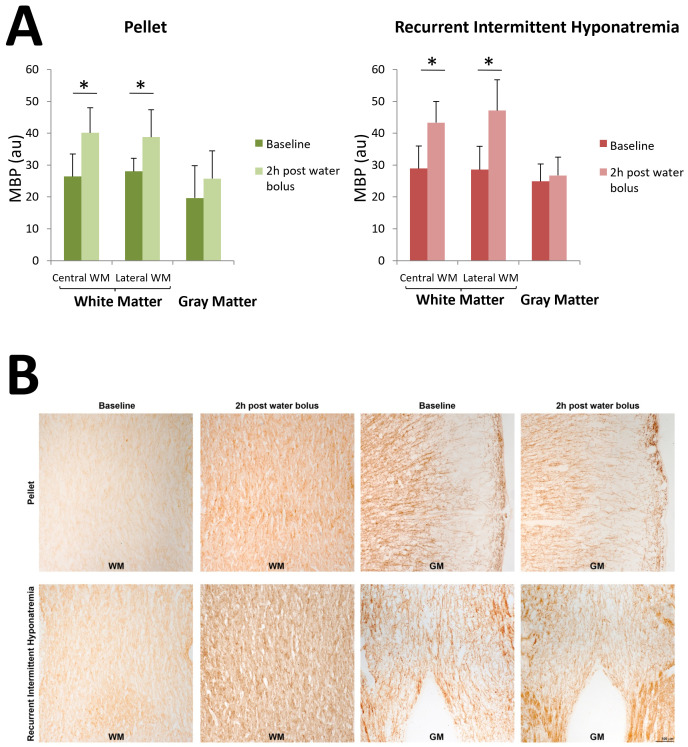
Effects of an intraperitoneal (i.p.) water overload equivalent to 10% of the animal’s body weight on the expression of myelin basic protein (MBP) compared to baseline (on day 15 of the study protocol, prior to the acute water overload). Results are expressed in arbitrary optical density units (au) as mean ± SD; n = 4 per experimental group. Panel A left: Pellet-fed animals. Factorial ANOVA of model p < 0.001; factor water bolus p < 0.001; factor region (central WM/lateral WM/GM) p < 0.001; interaction water bolus*region (central WM/lateral WM/GM) p < 0.001. *p < 0.05 for the comparison of central and lateral WM before and after water bolus administration. Panel A right: Recurrent intermittent hyponatremia (RIH) group (hyposodic diet + ddAVP/water). Factorial ANOVA of model p < 0.001; water bolus factor p < 0.001; region factor (central WM/lateral WM/GM) p < 0.001; interaction water bolus*region (central WM/lateral WM/GM) p = 0.003. *p < 0.05 for the comparison of central WM before and after a water bolus, and for the comparison of lateral WM before and after administration of a water bolus. Panel B: The images show: WM (corpus callosum) in pellet fed animals at baseline and 2h post water bolus; GM (motor cortex) in pellet fed animals at baseline and 2h post water bolus; WM (internal capsule internal capsule) under RIH at baseline and 2h post water bolus; and GM (hypothalamus) under RIH at baseline and 2h post water bolus. WM: white matter. GM: gray matter. RIH: recurrent intermittent hyponatremia.

Regarding MBP expression, this was increased in WM compared to GM, both in the RIH and the pellet-fed groups (p < 0.001 for both groups). Compared to pellet-fed animals, the induction of RIH tended to increase the global MBP expression (p = 0.06), but we failed to confirm such a trend in the separate analysis of the WM and the GM (Fig 4). Optical density values obtained for each of the regions studied separately are available in S4 Table.

### Acute hyponatremia and acute hyponatremia on recurrent intermittent hyponatremia

a) **Internal *milieu***

Two hours after i.p. administration of a water overload equivalent to 10% of the animal’s weight to the RIH group, a marked hypoosmolar hyponatremia was evident (baseline Na^+^_p_ 141.25 ± 0.96 vs final Na^+^_p_ 116.00 ± 1.16 mEq/L, p < 0.001; baseline Osm_p_ 294.05 ± 2.43 vs final Osm_p_ 261.73 ± 5.87 mOsm/kg, p = 0.001). Urine was not diluted (baseline Osm_u_ 201.25 ± 48.37 vs final Osm_u_ 364.87 ± 42.02 mOsm/kg, N.S.).

These results were similar to those found in the untreated pellet group, in which the animals were submitted to an analogous acute water overload (baseline Na^+^_p_ 140.25 ± 3.30 vs final Na^+^_p_ 113.33 ± 1.16 mEq/L, p < 0.001; baseline Osm_p_ 302.67 ± 11.55 vs final Osm_p_ 264.93 ± 9.15 mOsm/kg, p < 0.001; baseline Osm_u_ 736.00 ± 145.79 vs final Osm_u_ 592.48 ± 82.35 mOsm/kg, N.S.).

b) **Brain changes on MRI**

**Fig 5:**
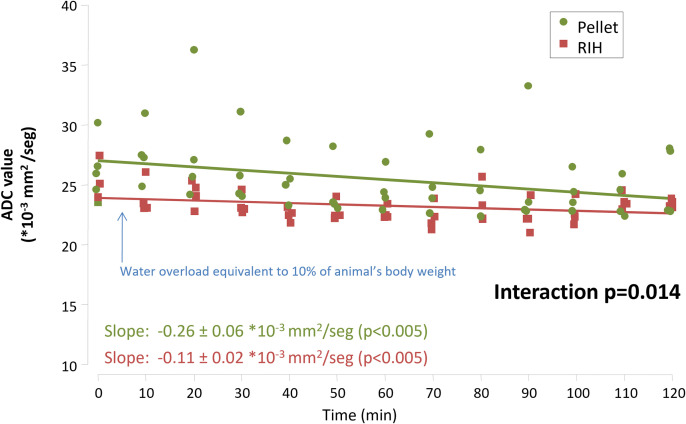
Evolution of apparent diffusion coefficient (ADC) values in the whole brain after a water overload equivalent to 10% of the animal’s body weight. n = 4 per group. Linear mixed model. RIH: Recurrent intermittent hyponatremia.

**Fig 6:**
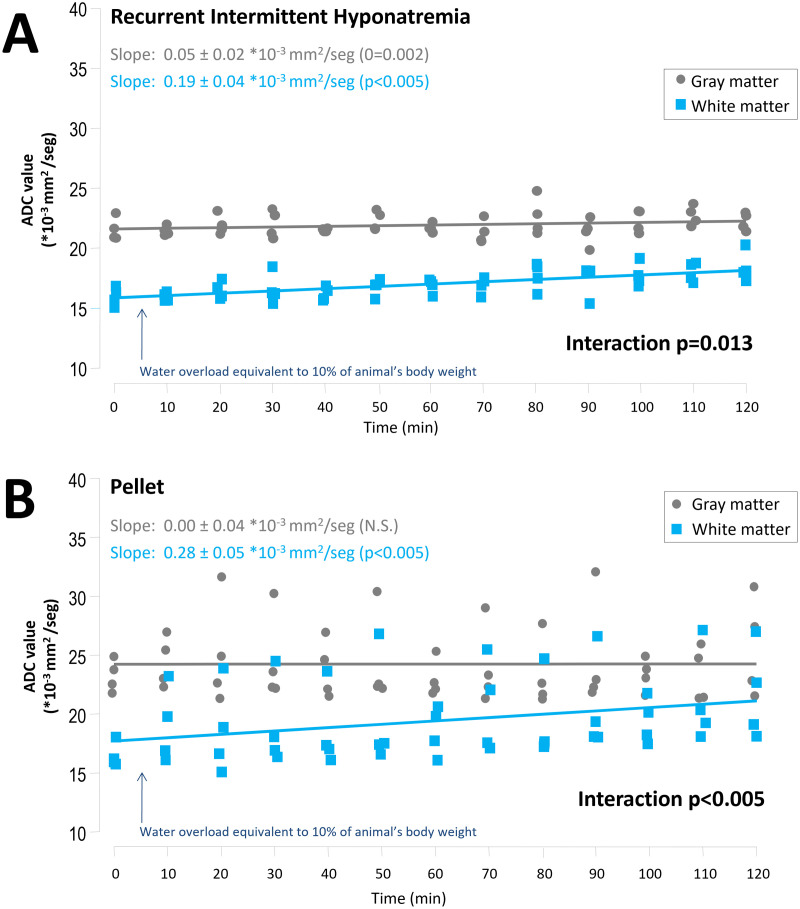
Evolution of apparent diffusion coefficient (ADC) in gray and white matter in both groups after a water overload equivalent to 10% of the animal’s weight. Panel A: Recurrent intermittent hyponatremia (hyposodic + ddAVP/water) group. n = 4 per group. Linear mixed model. Panel B: Pellet-fed group. n = 4 per group. Linear mixed model.

After the administration of the water overload to animals with RIH, we observed a progressive reduction in ADC values in the whole brain, with a decrease of 0.11 ± 0.02 mm^2^*10^3^/sec every 10 minutes (p < 0.001) (Fig 5). After excluding the effect CSF, the slope flattened, with a marginal decrease of 0.02 ± 0.01 mm^2^*10^3^/sec every 10 minutes (p = 0.055). The behaviour of the ADC slope was significantly different depending on whether CSF was considered (interaction p < 0.001) (S1 Fig). When analyzing separately GM and WM, both presented a progressive rise in ADC values, more marked in the WM (0.05 ± 0.02 and 0.19 ± 0.04 mm^2^*10^3^/sec every 10 minutes respectively, p < 0.001 for both; p = 0.013 for the interaction) (Fig 6A). Similarly, in the untreated pellet-fed animals we observed a progressive reduction in whole brain ADC values after the acute water overload, with a decrease of 0.26 ± 0.06 mm2*10^3^/sec every 10 minutes (p < 0.001) (Fig 5). Again, the slope flattened after excluding CSF, with a decrease of 0.11 ± 0.02 mm^2^*10^3^/sec every 10 minutes (p < 0.001) (S1 Fig). The behaviour of the ADC slope was significantly different depending on whether CSF was considered (interaction p = 0.012). When analyzing separately GM and WM, no significant changes were found in the behaviour of the GM over time, while the ADC value in the WM increased significantly by 0.28 ± 0.05 mm^2^*10^3^/sec every 10 minutes (p < 0.001) (Fig 6B).

The progressive reduction in ADC values observed in the whole brain was more pronounced in untreated pellet-fed animals compared to the RIH group (interaction p = 0.014 for whole brain, interaction p < 0.001 for whole brain without CSF) (Fig 5 and S1 Fig). The final values were not significantly different between the two groups, excluding or not CSF (S2 Table). It is noteworthy that the final mean ADC value in whole brain in untreated pellet-fed animals (25.21 ± 2.93 mm^2^*10^3^/sec) was similar to the baseline value in the RIH group (25.07 ± 1. 71 mm^2^*10^3^/sec).

c) **Brain changes on immunohistochemical analyses**

The i.p. administration of a water overload to the RIH group significantly increased the expression of GFAP at two hours post-injection in the central WM (p = 0.009) and the lateral WM (p = 0.008), without significant changes in the GM (Fig 3). In the pellet-fed group, an increased GFAP expression was only detected in the central WM (p < 0.001). Detailed analysis by regions is available in S3 Table Supplementary Material. The final situation (2h after water overload) did not show differences between the RIH and the pellet-fed group (Fig 3).

On the other hand, a significantly increased MBP expression was observed at two hours in the central (p < 0.001) and lateral (p < 0.001) WM, with no significant changes in the GM (Fig 4). Similar results were found in the pellet-fed group. In the lateral WM, a 65% increase in MBP expression was observed in the RIH compared to a 38% increase in the untreated pellet group (p = 0.05) (Fig 4, S4 Table).

## Discussion

The results of this work suggest that RIH, defined as a mild and transient hyponatremia that recovers within 24h of its induction but that occurs repeatedly over several consecutive days, induces water retention. Indeed, the brain water content is increased, especially in the GM. Astrogliosis occurs also in the GM.

As the half-life of ddAVP is 8-12h, it would be expected that the effects derived from its administration would recover 24h after administration, therefore explaining normalization of natremia at 24h after last injection.

In animals with RIH, water retention was evidenced by a reduction in diuresis and free water clearance and persistently low serum osmolality. This suggests that the organism is not able to completely eliminate the water overload even when serum sodium has already corrected. Kawakami et al. obtained results compatible with ours: switching from a pellet diet to a liquid diet associated with a continuous infusion of ddAVP, which induced chronic hyponatremia over 3 weeks, was shown to dilute the urine 5-fold and significantly decrease diuresis, secondary to an increased renal expression of aquaporin 2 [41]. The persistence of low osmolality in the presence of normal natremia requires further discussion. We found lower glucose levels in the RIH group, which could be due to the excess water retention and the animals’ inability to fully eliminate it. Given that the RIH group exhibits a negative electrolyte and caloric balance compared to the pellet fed group, translated into the observed weight loss, a catabolic state can be assumed to be present. Permanently reduced plasma osmolality may thus be assumed to result from a reduced plasma protein concentration. As plasma proteins are important in water balance over capillary walls, the observed tendency to cerebral edema in the RIH group may be caused by a permanently reduced oncotic pressure of plasma.

DWI is based on the movement of water molecules in tissues, which diffuse relatively freely and homogeneously in water-rich structures such as CSF or blood, while in tissues the mobility of water molecules is determined by the hystoarchitecture, leading to higher ADC values in GM as compared to WM, where the directionality of axons restricts water movement to a preferential direction along the fibres (anisotropic diffusion). Quantitative representation of water diffusion is conveyed by the ADC for the different voxels, usually presented as ADC maps [42]. Typically, cellular edema results in a restriction of diffusion associated with a decrease in ADC, while increases in extracellular water are commonly associated to increased ADC values [43,44]. As for the whole-brain ADC value, this may be more challenging to interpret as it combines weighted contributions of GM, WM, CSF and blood.

In our study, we found a lower ADC value in WM compared to GM, as expected. Our data are consistent with those reported by Kuroiwa et al. [45] in cats. In a model of vasogenic edema due to cryogenic brain damage, these authors demonstrated a difference in the hystoarchitectural properties of GM and WM that accounts for the lower values of ADC in WM [42,46,47].

In the RIH group the baseline ADC value in the whole brain was significantly lower than in pellet-fed animals, regardless of whether brain CSF was accounted for or not. This finding may suggest an overall increase of intracellular water in these animals’ brains, seemingly secondary to the water retention discussed above. This finding has not been previously described in the literature, to the best of our knowledge, since it is generally assumed that the correction of acute hyponatremia in the first hours has no lasting consequences [7]. When analyzing separately the different regions of the brain, we observed that the ADC value was lower in the RIH group than in pellet-fed animals in the GM, but there were no differences in the WM between the two groups. These results suggest that the excess water accumulated in RIH is mainly located in the GM, presumably within the astrocytes. Although neurologic dysfunction in hyponatremia is secondary to the movement of water into brain cells driven by an osmotic gradient, astrocytes are the most sensitive and rapid responders to osmotic changes. Aquaporin 4 (AQP4), a passive water channel, is predominantly expressed in the pericapillary foot processes of astrocytes [48–56]. Indeed, classic electron microscopy studies suggested a relative sparing of the neuronal component compared to the predominant swelling of astrocytes [57–59]. However, we do not exclude the involvement of other contributing mechanisms such as neuronal edema.

RIH induces hyponatremia only for a few hours daily. This raises the question of whether such brief daily episodes are sufficient to induce an adaptive response in the brain like that observed in chronic hyponatremia. To assess the potential adaptation of the brain to RIH, we conducted localized *in-vivo* MR spectroscopy. A reduction in the concentration of certain osmolytes (myo-inositol, choline, creatine, NAA) in various brain regions has been described as an adaptation to chronic hyponatremia, to restore brain volume [60]. We chose caudate-putamen as the study region because it was easy to identify, deep and of convenient size. No significant differences in osmolyte quantification were observed in animals with RIH compared to controls. Therefore, in our experiment we did not observe a brain adaptation analogous to that reported in chronic hyponatremia. Anyhow, we cannot rule out that *ex-vivo* spectroscopy [61] or larger studies, including different brain regions, might lead to different results, since the caudate-putamen could not be representative enough of the behaviour of the global brain.

In the RIH model we found an increase in the expression of GFAP in GM compared to pellet-fed animals. This result, together with the lower initial ADC values observed in the GM of this group, suggests that few hours of hyponatremia per day may be sufficient to induce water accumulation in GM astrocytes, which could contribute to explain the astrogliosis observed.

While changes in myelin are often attributed to oligodendrocyte dysfunction, several studies indicate that astrocytes are also involved in the regulation of both myelination and remyelination. Astrocytes are connected to each other and to oligodendrocytes by connexins and function as a glial syncytium whose integrity is crucial for CNS functions and for the maintenance of the myelin sheath [62–67]. It has been shown that astrocytes can modulate myelin regeneration through metabolic pathways such as cholesterol biosynthesis and oxidative stress regulation via the Nrf2 pathway. Sustained activation of Nrf2 in astrocytes can inhibit remyelination, whereas its inhibition or stimulation of astrocytic lipid metabolism can restore it [68]. Therefore, we consider that the astrocytic dysfunction observed in our study may indirectly contribute to oligodendrocyte dysfunction [69]; and we wanted to explore if the damage in astrocytes could have an early impact on oligodendrocytes or myelin formation. In our experiment, no change in MBP expression was observed in RIH compared to controls. This result is consistent with the absence of significant differences in the initial ADC value in the WM in both experimental groups. This fact probably reflects that RIH does not induce oligodendrocyte injury, or perhaps the observation time was too short to detect changes in MBP expression in the WM.

In RIH, a progressive and significant decrease in whole brain ADC values was observed over the first two hours after the administration of a water overload. Compared to what was observed after the same procedure in pellet-fed animals, the ADC decline was significantly slower. This was true with and without including the contribution of CSF. The differences between the two groups tended to disappear over time, reaching a final ADC value very similar between them. This suggests that the entry of water into the intracellular space can be slower in RIH. A possible explanation could be the induction of post-transcriptional changes in osmolyte transporters, increasing osmolyte extrusion to the extracellular space and consequently decreasing the entry of water into the cell, with a mechanism analogous to that already described in hypertonicity [70], although these changes have not been reported in hypotonic conditions. Another possibility is that the RIH group undergoes a higher rate of regulatory volume decrease, leading to a smaller increase in volume. Finally, a down-regulation of AQP4 in this situation could also explain a slower entry of water into the cells, as it has been described in other brain edema models [15,71–73].

Another mechanism that can play a role in this model is the existence of an initial displacement of blood and CSF outside the skull secondary to an early increase in intracellular volume in RIH. After excluding the effect of CSF, a significant decline in ADC was still present in the control group, suggesting that, despite extrusion of CSF outside the skull, a degree of intracellular edema developed anyhow. However, in the RIH group, after excluding the effect of CSF, the change in slope was marginal (p = 0.055), suggesting a smaller increase in intracellular edema. This goes against our initial hypothesis that this group would be more severely affected by a water challenge [74]. Our ADC measurements in GM and WM ROIs are not influenced by CSF, as the ROIs selected did not include any CSF.

When analyzing the WM separately in the RIH group, we observed a small but significant increase in ADC (slope 0.05 ± 0.02 *10^-3^mm^2^/sec), which did not differ from that observed in pellet-fed controls, where the slope did not change significantly. Therefore, the pathophysiological relevance of this slight ADC increase remains to be confirmed. A progressive rise in ADC values in the WM was also observed in both groups, in accordance with a differential response to hypotonicity between astroglia and oligodendroglia, as proposed by some authors [75,76]. In a cat model of acute ischemia, water accumulation was observed within axons, along with an expansion of the periaxonal space in myelinated fibers [46]. A predominantly extracellular water accumulation in WM, as described in that study, could account for our findings (progressive ADC increase in WM following water overload).

In both the RIH and the pellet-fed groups the water overload induced an increase in the expression of GFAP in the WM compared to baseline, without significant changes in the GM. These results taken together suggest that the administration of a water overload produces an activation of macroglia, specifically astrocytes, in the WM but not in the GM.

As mentioned above, the water overload in the RIH group induced a small increase in ADC values in the GM throughout the observation period, which did not reflect in a change in GFAP expression. It is possible that the astrocyte volume change in this case is not sufficient to induce astrogliosis.

Our study has several limitations. Firstly, we only used female rats. It is known that females are more sensitive to hyponatremia-induced changes [25,26,77,78], which explains the existence of several studies in animal models focused only on females [74,79,80]. Although this choice limits the generalizability of our results, it increases homogeneity, which is an advantage given of our reduced sample size. It would be advisable in the future to study potential sex-related differences in the brain response to RIH. As our goal was to develop a new experimental model, RIH, we aimed to minimize the number of animals used in this proof-of-concept study in accordance with the 3Rs principle. Secondly, direct measurements of brain water content were not performed. While classic works involved weighting extracted brain tissue before and after desiccation or measuring the specific gravity of brain fragments [34,81,82], our experimental design focused on the use of ADC to estimate brain water content, as Lin et al. demonstrated a good correlation between MRI image data and wet/dry measurements [83]. This approach offered the distinct advantage of enabling longitudinal assessment of water content changes in different brain regions separately. Our experimental design did not include protein measurements, as the animals required daily blood draws to monitor natremia. The volume of blood obtained was deliberately minimal and analyzed using a gas analyzer, which did not provide protein or albumin measures, to limit blood loss and avoid potential hypovolemia. Some authors propose that intraperitoneal administration of a water overload induces a redistribution of sodium toward the peritoneal fluid, which has become hypotonic relative to the extracellular space, leading to a reduction in plasma volume and hemoconcentration [79]. This situation could potentially lead to an increased secretion of endogenous ADH, as suggested by the authors, which could in turn lead to renal water retention and possibly hyponatremia. Although this mechanism must indeed be taken into consideration when interpreting our findings, we believe all of our findings are consistent with brain water accumulation. Finally, the lack of a control group with RIH but not subjected to an acute water overload limits the interpretation of our findings in GFAP and MBP expression, as mechanisms other than hyponatremia itself could be implicated. If our findings are confirmed, other relevant topics could be explored with this model, such as AQP4 expression in astrocytes [84], connexins 43 and 47 expression—responsible for tight-junctions between astrocytes and oligodendrocytes and necessary for proper myelin function [85] —, or renal AQP2 expression as a possible mechanism involved in the water retention [41]. MRI studies do not provide information at a cellular level, so we can only assume that changes in ADC do in fact reflect net movement of water between extracellular and intracellular compartments. In addition, the limited spatial resolution of DWI must be noted, especially for some ROIs such as the hypothalamus. Immunohistochemical analysis allowed us to determine the involvement of astrocytes and oligodendrocytes, the cell types that, according to the literature, would play a greater role in these situations. This exploratory study targeted some ROIs, but deepening in the potential differences between regions would be desirable. In particular, special attention should be paid to the hippocampus and cerebellum, both regions highly involved in learning, memory and motor functions. Likewise, it would be interesting to use other techniques for the assessment of cell morphology, such as electron microscopy or multiphoton laser scanning microscopy [80,86,87].

We have successfully developed and characterized a novel animal model of Recurrent Intermittent Hyponatremia, a condition not previously described. Remarkably, our findings reveal that just a few hours of daily hyponatremia—when repeated over time—are sufficient to induce brain edema, even after 24-hour normalization. Unlike existing models that focus on severe acute or chronic hyponatremia, which are uncommon in clinical practice, our model highlights the often-overlooked role of intermittent mild hyponatremia as a significant contributor to disease. This breakthrough opens exciting avenues for research and emphasizes its potential implications for clinical practice.

## Supporting information

S1 Fig Evolution of apparent diffusion coefficient (ADC) values in the whole brain with and without the contribution of cerebrospinal fluid after a water overload equivalent to 10% of the animal’s body weight. n = 4 per group.
(TIF)

S1 Table Total densitometry quantification area of the different regions under study. (DOCX)

S2 Table ADC values at baseline and 120 minutes after a water overload equivalent to 10% of the animal’s body weight in the different experimental groups. Results are expressed as mean ± SD; n = 4 per experimental group. RIH: recurrent intermittent hyponatremia. WB: whole brain; WB w/o CSF: whole brain discounting the effect of cerebrospinal fluid; GM: gray matter; CP: caudate-putamen; Cx: cortex; HC: hippocampus; HT: hypothalamus; WM: white matter. p-value for the comparison between experimental groups at baseline and after the water overload. Bold figures indicate statistical significance.
(DOCX)

S3 Table Detailed analysis of glial fibrillary acidic protein (GFAP) expression in the different regions in the pellet-fed and recurrent intermittent hyponatremia (RIH) groups before and after an intraperitoneal bolus of water equivalent to 10% of the animal’s weight. Results expressed in arbitrary units of optical density, as mean ± SD; n = 4 per experimental group. *p < 0.05 compared to the baseline pellet-fed group. $ p < 0.05 compared to the baseline RIH. † p < 0.05 compared to the pellet-fed group after the water bolus.
(DOCX)

S4 Table Detailed analysis of myelin basic protein (MBP) expression in the different regions in the pellet-fed and recurrent intermittent hyponatremia (RIH) groups before and after an intraperitoneal bolus of water equivalent to 10% of the animal’s weight. Results expressed in arbitrary units of optical density, as mean ± SD; n = 4 per experimental group. *p < 0.05 compared to the baseline pellet-fed group. $ p < 0.05 compared to the baseline RIH. † p < 0.05 compared to the pellet-fed group after the water bolus.
(DOCX)

S1 Dataset Internal milieu. (XLSX)

S2 Dataset Brain changes on MRI. (XLSX)

S3 Dataset Brain changes on immunohistochemical analyses. (XLSX)
