# Supplementary figures and images for: Recurrent intermittent hyponatremia: A new experimental model
Source: PLoS One. 2026 Feb 20;21(2):e0341743. doi: 10.1371/journal.pone.0341743 (PMC12922978; doi:10.1371/journal.pone.0341743)

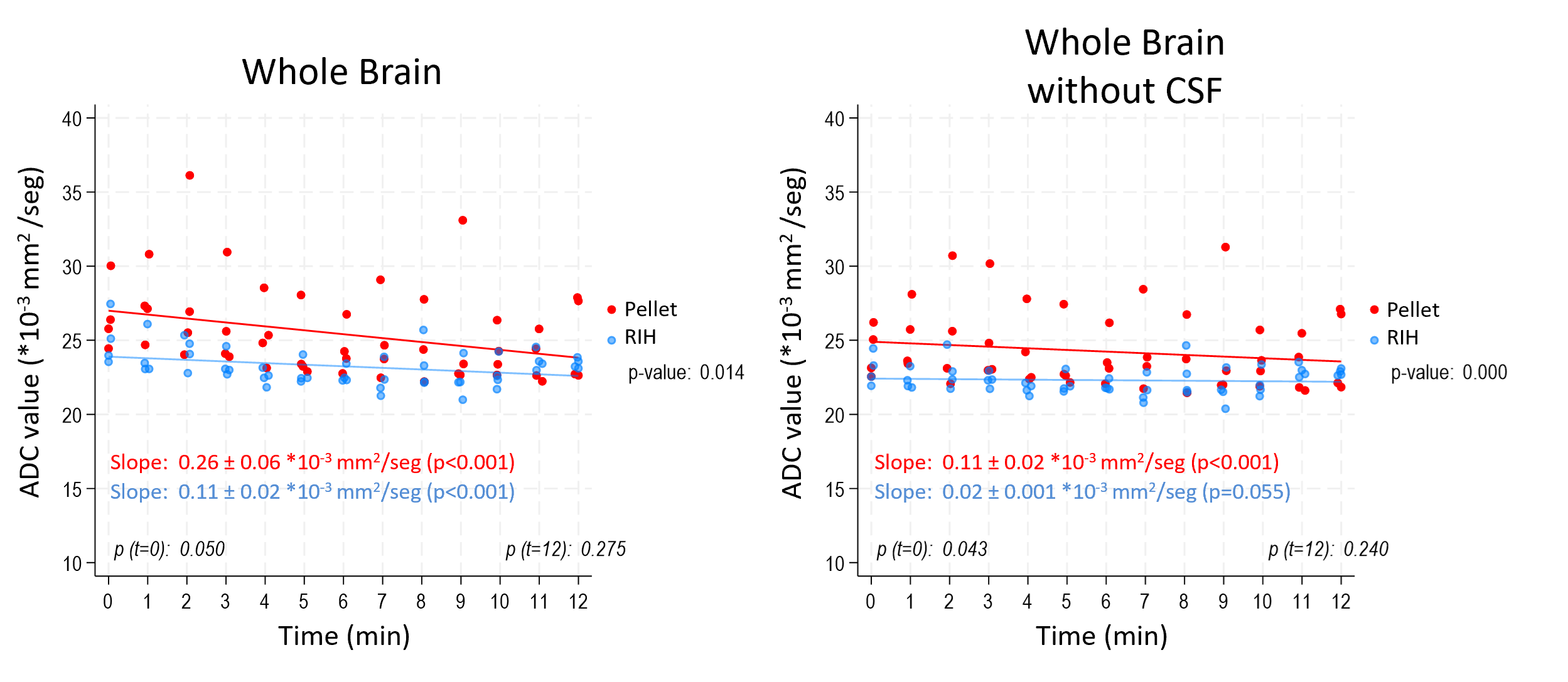

Supplement: S1 Fig — n = 4 per group. (TIF) [file pone.0341743.s001.tif]
