# Supplementary material for: Recurrent intermittent hyponatremia: A new experimental model
Source: PLoS One. 2026 Feb 20;21(2):e0341743. doi: 10.1371/journal.pone.0341743 (PMC12922978; doi:10.1371/journal.pone.0341743)
Supplement: S1 Table — (DOCX) [file pone.0341743.s002.docx]

| Region | Densitometry area (μm^2^) |
| --- | --- |
| Central rostral corpus callosum | 2864736 |
| Central mid corpus callosum | 711888 |
| Central caudal corpus callosum | 711888 |
| Right lateral corpus callosum | 1032238 |
| Left lateral corpus callosum | 1032238 |
| Internal capsule | 1521774 |
| Motor cortex | 1448 |
| Periventricular hypothalamus | 1923 |

S1 Table. Total densitometry quantification area of the different regions under study.
