# Supplementary material for: Recurrent intermittent hyponatremia: A new experimental model
Source: PLoS One. 2026 Feb 20;21(2):e0341743. doi: 10.1371/journal.pone.0341743 (PMC12922978; doi:10.1371/journal.pone.0341743)
Supplement: S2 Table — Results are expressed as mean ± SD; n = 4 per experimental group. RIH: recurrent intermittent hyponatremia. WB: whole brain; WB w/o CSF: whole brain discounting the effect of cerebrospinal fluid; GM: gray matter; CP: caudate-putamen; Cx: cortex; HC: hippocampus; HT: hypothalamus; WM: white matter. p-value for the comparison between experimental groups at baseline and after the water overload. Bold figures indicate statistical significance. (DOCX) [file pone.0341743.s003.docx]

| Region | Baseline ADC  (mm^2^ *10^3^/seg) | | p | Final ADC  (mm^2^ *10^3^/seg) | | p |
| --- | --- | --- | --- | --- | --- | --- |
|  | **Pellet** | **RIH** |  | **Pellet** | **RIH** |  |
| WB | 26.71 ± 2.40 | 25.07 ± 1.71 | **0.05** | 25.21 ± 2.93 | 23.43 ± 0.38 | 0.275 |
| WB w/o CSF | 24.29 ± 1.66 | 23.1 ± 1.06 | **0.043** | 24.45 ± 2.85 | 22.81 ± 0.24 | 0.240 |
| GM | 23.45 ± 1.33 | 21.63 ± 0.95 | **0.054** | 25.80 ± 4.27 | 22.20 ± 0.79 | 0.137 |
| · CP | 23.32 ± 1.00 | 21.28 ± 0.91 | **0.047** | 26.71 ± 5.15 | 22.25 ± 1.73 | **0.044** |
| · Cx | 23.70 ± 1.90 | 21.70 ± 1.16 | 0.092 | 26.11 ± 4.98 | 22.26 ± 0.96 | 0.239 |
| · HC | 23.28 ± 1.43 | 23.00 ± 1.68 | 0.068 | 23.51 ± 4.64 | 21.97 ± 0.62 | 0.357 |
| HT | 25.34 ± 2.80 | 24.97 ± 1.30 | 0.279 | 23.45 ± 3.34 | 23.81 ± 0.76 | 0.743 |
| WM | 16.52 ± 1.07 | 16.02 ± 0.75 | 0.192 | 21.70 ± 4.04 | 18.40 ± 1.33 | **0.043** |

S2 Table. ADC values at baseline and 120 minutes after a water overload equivalent to 10% of the animal's body weight in the different experimental groups. Results are expressed as mean ± SD; n=4 per experimental group. RIH: recurrent intermittent hyponatremia. WB: whole brain; WB w/o CSF: whole brain discounting the effect of cerebrospinal fluid; GM: gray matter; CP: caudate-putamen; Cx: cortex; HC: hippocampus; HT: hypothalamus; WM: white matter. p-value for the comparison between experimental groups at baseline and after the water overload. Bold figures indicate statistical significance.
