# Supplementary material for: Recurrent intermittent hyponatremia: A new experimental model
Source: PLoS One. 2026 Feb 20;21(2):e0341743. doi: 10.1371/journal.pone.0341743 (PMC12922978; doi:10.1371/journal.pone.0341743)
Supplement: S3 Table — Results expressed in arbitrary units of optical density, as mean ± SD; n = 4 per experimental group. *p < 0.05 compared to the baseline pellet-fed group. $ p < 0.05 compared to the baseline RIH. † p < 0.05 compared to the pellet-fed group after the water bolus. (DOCX) [file pone.0341743.s004.docx]

| **Region** | **Pellet** | | **RIH** | |
| --- | --- | --- | --- | --- |
| **(mean ± SD)** | **Baseline** | **2h post water bolus** | **Baseline** | **2h post water bolus** |
| Central rostral corpus callosum | 12.05 ± 2.55 | 19.50 ± 5.55 | 13.27 ± 4.17 | 24.80 ± 3.72 $ |
| Central mid corpus callosum | 11.62 ± 1.92 | 23.54 ± 2.51 * | 11.89 ± 1.21 | 15.37 ± 3.34 † |
| Central caudal corpus callosum | 9.87 ± 2.21 | 21.00 ± 4.05 * | 18.95 ± 1.31 * | 20.54 ± 3.98 |
| Lateral right corpus callosum | 22.34 ± 2.43 | 25.56 ± 1.91 | 22.98 ± 2.21 | 27.18 ± 3.09 |
| Lateral left corpus callosum | 20.61 ± 5.82 | 22.23 ± 1.86 | 12.84 ± 4.12 | 26.42 ± 3.92 $ |
| Internal capsule | 20.91 ± 1.45 | 17.77 ± 6.99 | 21.08 ± 3.35 | 24.68 ± 10.62 |
| Motor cortex | 25.25 ± 1.83 | 30.15 ± 6.70 | 33.20 ± 7.16 | 38.41 ± 11.78 |
| Periventricular hypothalamus | 24.90 ± 3.00 | 32.28 ± 2.14 * | 33.24 ± 3.63 * | 35.24 ± 3.52 |

S3 Table. Detailed analysis of glial fibrillary acidic protein (GFAP) expression in the different regions in the pellet-fed and recurrent intermittent hyponatremia (RIH) groups before and after an intraperitoneal bolus of water equivalent to 10% of the animal's weight. Results expressed in arbitrary units of optical density, as mean ± SD; n=4 per experimental group. *p<0.05 compared to the baseline pellet-fed group. $ p<0.05 compared to the baseline RIH. † p<0.05 compared to the pellet-fed group after the water bolus.
