# Supplementary material for: Recurrent intermittent hyponatremia: A new experimental model
Source: PLoS One. 2026 Feb 20;21(2):e0341743. doi: 10.1371/journal.pone.0341743 (PMC12922978; doi:10.1371/journal.pone.0341743)
Supplement: S4 Table — Results expressed in arbitrary units of optical density, as mean ± SD; n = 4 per experimental group. *p < 0.05 compared to the baseline pellet-fed group. $ p < 0.05 compared to the baseline RIH. † p < 0.05 compared to the pellet-fed group after the water bolus. (DOCX) [file pone.0341743.s005.docx]

| **Region** | **Pellet** | | **RIH** | |
| --- | --- | --- | --- | --- |
| **(mean ± SD)** | **Baseline** | **2h post water bolus** | **Baseline** | **2h post water bolus** |
| Central rostral corpus callosum | 18.29 ± 4.18 | 45.04 ± 8.43 * | 23.71 ± 5.55 | 42.80 ± 4.51 $ |
| Central mid corpus callosum | 31.53 ± 2.32 | 38.35 ± 8.14 | 30.80 ± 7.55 | 39.73 ± 3.43 |
| Central caudal corpus callosum | 29.46 ± 5.15 | 37.07 ± 6.33 | 32.30 ± 6.05 | 47.34 ± 9.49 $ |
| Lateral right corpus callosum | 26.98 ± 2.40 | 40.31 ± 4.44 * | 33.67 ± 4.79 | 51.25 ± 5.26 $† |
| Lateral left corpus callosum | 28.01 ± 5.78 | 45.86 ± 5.35 * | 30.25 ± 5.53 | 48.05 ± 3.36 $ |
| Internal capsule | 29.15 ± 4.29 | 30.17 ± 7.18 | 21.83 ± 6.78 | 42.03 ± 15.59 $ |
| Motor cortex | 28.24 ± 5.47 | 20.85 ± 6.60 | 28.12 ± 5.31 | 22.33 ± 3.42 |
| Periventricular hypothalamus | 11.09 ± 3.53 | 30.64 ± 8.27 * | 21.67 ± 3.49 | 31.13 ± 3.86 |

S4 Table. Detailed analysis of myelin basic protein (MBP) expression in the different regions in the pellet-fed and recurrent intermittent hyponatremia (RIH) groups before and after an intraperitoneal bolus of water equivalent to 10% of the animal's weight. Results expressed in arbitrary units of optical density, as mean ± SD; n=4 per experimental group. *p<0.05 compared to the baseline pellet-fed group. $ p<0.05 compared to the baseline RIH. † p<0.05 compared to the pellet-fed group after the water bolus.
